# Awareness of and willingness to use oral pre-exposure prophylaxis (PrEP) for HIV prevention among sexually active adults in Malawi: results from the 2020 Malawi population-based HIV impact assessment

**DOI:** 10.1186/s12879-023-08683-1

**Published:** 2023-10-20

**Authors:** Alinune Nathanael Kabaghe, Victor Singano, Danielle Payne, Alice Maida, Rose Nyirenda, Kelsey Mirkovic, Andreas Jahn, Pragna Patel, Kristin Brown, Mansoor Farahani, Felix Kayigamba, Lyson Tenthani, Francis Ogollah, Andrew Auld, Fatima Zulu, Wezi Msungama, Nellie Wadonda-Kabondo

**Affiliations:** 1 Division of Global HIV and TB, Centers for Disease Control and Prevention, Lilongwe, Malawi; 2 Department of HIV, AIDS and Hepatitis, Ministry of Health, Lilongwe, Malawi; 3 Division of Global HIV and TB, Centers for Disease Control and Prevention, Atlanta, United States of America; 4 ICAP Headquarters, Columbia University, New York, United States of America; 5 ICAP in Malawi, Lilongwe, Malawi; 6 Centers for Disease Control and Prevention, US Embassy - Lilongwe, 2nd Floor NICO House, P.O. Box 30016, Lilongwe, Malawi

**Keywords:** HIV, PrEP, HIV prevention, Malawi, Population survey

## Abstract

**Background:**

The World Health Organization recommends Pre-Exposure Prophylaxis (PrEP) for all populations at substantial risk of HIV infection. Understanding PrEP awareness and interest is crucial for designing PrEP programs; however, data are lacking in sub-Saharan Africa. In Malawi, oral PrEP was introduced in 2018. We analyzed data from the 2020 Malawi Population-based HIV Impact Assessment (MPHIA) to assess PrEP awareness and factors associated with PrEP interest in Malawi.

**Methods:**

MPHIA 2020 was a national cross-sectional household-based survey targeting adults aged 15 + years. Oral PrEP was first described to the survey participants as taking a daily pill to reduce the chance of getting HIV. To assess awareness, participants were asked if they had ever heard of PrEP and to assess interest, were asked if they would take PrEP to prevent HIV, regardless of previous PrEP knowledge. Only sexually active HIV-negative participants are included in this analysis. We used multivariable logistic regression to assess sociodemographic factors and behaviors associated with PrEP interest. All results were weighted.

**Results:**

We included 13,995 HIV-negative sexually active participants; median age was 29 years old. Overall, 15.0%, 95% confidence interval (CI): 14.2–15.9% of participants were aware of PrEP. More males (adjusted odds ratio (aOR): 1.3, 95% CI: 1.2–1.5), those with secondary (aOR: 1.5, 95% CI: 1.2-2.0) or post-secondary (aOR: 3.4, 95% CI: 2.4–4.9) education and the wealthiest (aOR: 1.6, 95% CI: 1.2-2.0) were aware of PrEP than female, those without education and least wealthy participants, respectively. Overall, 73.0% (95% CI: 71.8–74.1%) of participants were willing to use PrEP. Being male (aOR: 1.2; 95% CI: 1.1–1.3) and having more than one sexual partner (aOR: 1.7 95% CI: 1.4–1.9), were associated higher willingness to use PrEP.

**Conclusions:**

In this survey, prior PrEP knowledge and use were low while PrEP interest was high. High risk sexual behavior was associated with willingness to use PrEP. Strategies to increase PrEP awareness and universal access, may reduce HIV transmission.

## Background

Pre-exposure prophylaxis (PrEP) reduces the risk of HIV acquisition and recommended by World Health Organization (WHO) for individuals at substantial risk of HIV acquisition [1]. PrEP is currently available as an oral pill taken once daily to prevent the risk of HIV acquisition while several alternative PrEP products are in the pipeline [2, 3]. The WHO recommends PrEP to be offered in combination with other prevention services: condom and lubricant promotion, post-exposure prophylaxis (PEP), voluntary medical male circumcision (VMMC), risk reduction education, harm reduction, and other structural interventions to reduce vulnerability to HIV infection. For women, PrEP can be empowering and within their control as it can be taken without requiring consent or negotiation with partners unlike other interventions such as condom use [4].

In Malawi, PrEP roll out started in 2018 for individuals at substantial HIV risk but there is currently limited information on the awareness and willingness to use and access the intervention in the general population [5]. Most published studies on awareness, interest and use of PrEP have been among specific high-risk or priority populations such as men who have sex with men (MSM), people who inject drugs, transgender persons, and female sex workers [6, 7]. There are several qualitative studies in priority populations in Malawi in defined geographic areas and include a small sample size of female sex workers [8], adolescent girls and young women (AGYW) [9, 10] and men who have sex with men (MSM) [11]. Findings from these studies demonstrated that most participants were unaware of PrEP but were interested to use PrEP for HIV prevention. Malawi and other countries aiming to achieve epidemic control are scaling up PrEP beyond key and priority populations. PrEP reduces HIV transmission in the general population [12, 13].

To inform PrEP programming and contribute to achieving HIV epidemic control in Malawi and similar settings, there is need to understand the current levels of awareness and willingness to use PrEP as well as factors associated with awareness and willingness to use PrEP in the general population. In this context, we analyzed data from a nationally representative survey to assess oral PrEP awareness and willingness and factors associated with awareness of and willingness to use PrEP among adults in Malawi.

## Methods

### Study design, setting and population

We analyzed data collected from the Malawi Population-based Impact Assessment (MPHIA) 2020–2021 [14]. The MPHIA was a cross-sectional, two-stage, cluster-randomized nationally representative household survey conducted in Malawi in adults aged 15 + years old. The primary objective of MPHIA was to estimate the subnational prevalence of viral load suppression and the progress towards the UNAIDS 95-95-95 goals among the adults aged 15+. The survey was implemented by the Malawi Ministry of Health and ICAP at Columbia University with funding from the President’s Emergency Plan for AIDS Relief (PEPFAR) and technical support from US Centers for Disease Control and Prevention (CDC) between January 2020 and April 2021.

### Data Collection

Trained interviewers administered household and individual questionnaires to participants on mobile tablet devices with Census and Survey Processing System (CSPro) software. The individual interviews included questions on demographics, sexual behaviors, HIV testing and treatment history. HIV counselling and testing was conducted using Malawi’s HIV national testing algorithm and results provided to the participants on the same day. Data for PrEP elements were collected by first describing PrEP to the survey participants as “a process of taking a daily pill to reduce the chance of getting HIV”. This definition was valid for the PrEP modality available in the country at the time. To assess awareness, the participants were asked if they had ever heard of PrEP prior to the survey and asked if they had ever been offered PrEP (if they had heard of PrEP). The participants that were offered PrEP were then asked if they had taken or were still taking PrEP. Lastly, to assess interest, all the participants were asked if they would take PrEP to prevent HIV if they were offered. In addition, participants were asked to report on whether they had sex in the prior 12 months, the number of sexual partners in the past 12 months and for their last three sexual partners, whether they had used a condom. Participants were asked whether all partners in the past 12 months were a spouse/live-in partner, whether they were using any contraceptive, and whether they had accessed an HIV test in the prior 12 months.

### Statistical analysis

Data from the survey was analyzed using STATA 16 (StataCorp LLC) accounting for the survey design. In this analysis, we only included data from (i) participants whose HIV test result was negative and were therefore eligible to receive PrEP and (ii) reported being sexually active in the preceding 12 months. We calculated weighted proportions of participants aware of, offered, and willing to use PrEP and estimated 95% confidence intervals (CI) using Jackknife variance estimation. We also conducted a bivariate logistic regression analysis for the association between willingness to use PrEP and demographic variables, reported number of sexual partners, and HIV testing in the prior 12 months. We fitted a multivariable logistic regression model to assess how awareness of and willingness to use PrEP were associated with *a priori* variables (age, sex, education level, residence, geographic location, marital status, sexual risk, and HIV testing). The *a priori* variables were selected given their biological and behavioral importance in relation to PrEP in previous literature.

### Ethical considerations

The study protocol was reviewed and approved by the Institutional Review Boards of CDC, Columbia University, and the National Health Research Ethics Committee in Malawi. All participants aged 18 + years provided consent to participate while those 15–17 years old assented and had legal guardian approval.

## Results

### Demographic and clinical characteristics of the participants

**Fig. 1:**
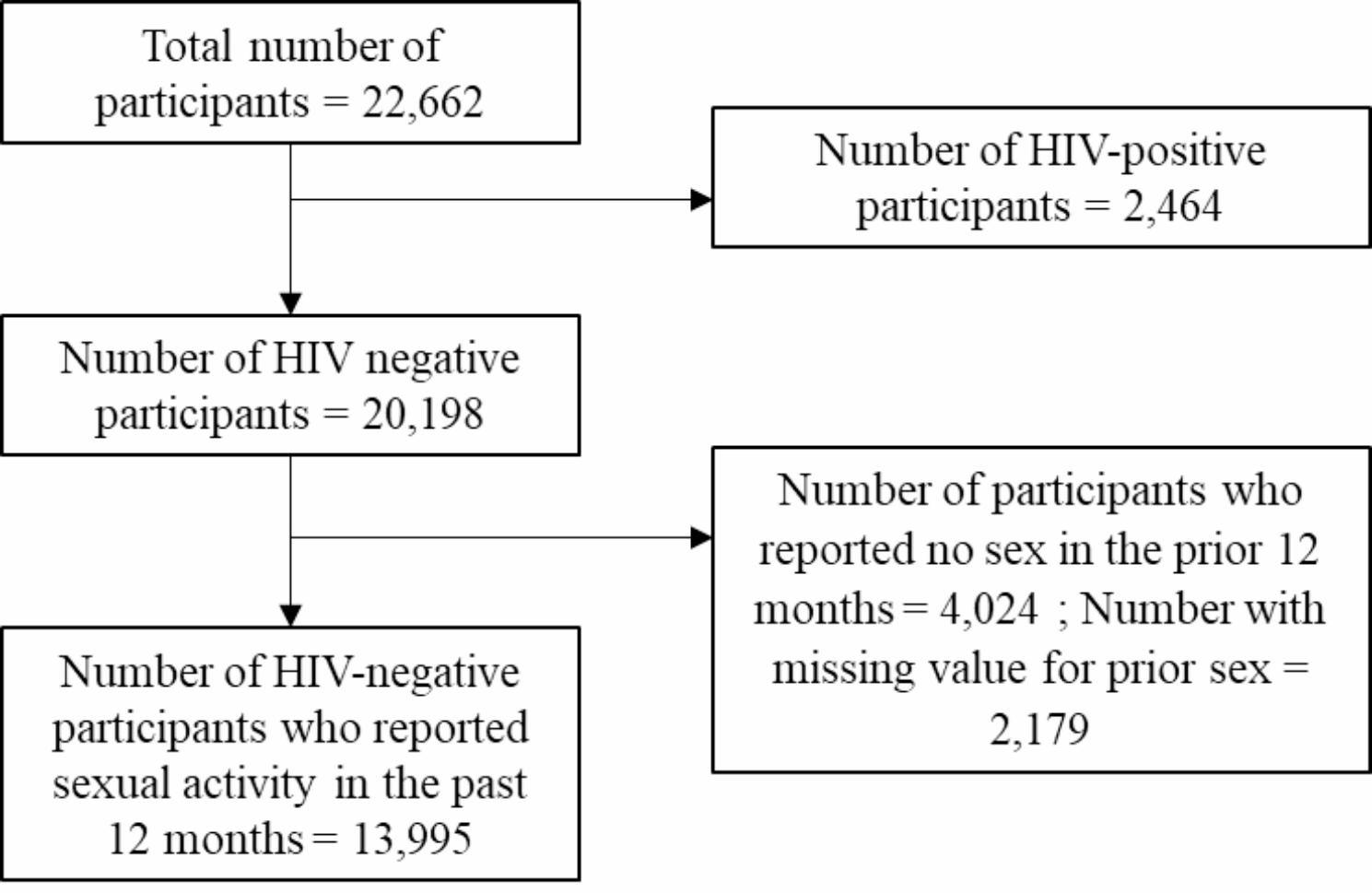
Flow diagram of participants in MPHIA 2020-21 survey and included in the analysis

A total of 12,815 households and 26,519 participants 15 + years old were interviewed; of these, 22,662 had HIV test results and 20,198 had an HIV-negative result (Fig. 1). Of the HIV-negative participants, 13,995 reported sexual activity in the prior 12 months.

The median age of the sexually active HIV-negative participants was 29 years old (IQR: 22–40 years old) (Table 1). Most participants were aged 20–24 years old (19.9%) or 25–29 years old (17.5); 14.7% of males reporting sexual activity in the prior 12 months were 50 + years, compared to 9.4% of females in the same age category. The highest level of education reported by most participants was primary school (61.6%) although the proportion of females who had no education (11.2%) was higher than males who had no education (6.4%). Conversely, more men (30.6%) had attended secondary education than women (21.0%). Most participants (73.0%) were married/cohabiting.

**Table 1:** Demographic characteristics of sexually active HIV-negative participants from MPHIA 2020–2021 household survey

Participant characteristic	Males: n (weighted %)	Female: n (weighted %)	Total: n (weighted % or interquartile range)
Total	6,432 (51.3)	7,563 (48.7)	13,995
Median age in years	30 (23–42)	28 (22–38)	29 (22–40)
Age categories in years			
15–19	826 (12.8)	887 (14.5)	1,713 (13.6)
20–24	1,174 (18.2)	1,718 (21.7)	2,892 (19.9)
25–29	910 (16.4)	1,413 (18.6)	2,323 (17.5)
30–34	774 (13.7)	965 (13.6)	1,739 (13.6)
35–39	693 (10.4)	884 (10.0)	1,577 (10.2)
40–44	538 (7.7)	564 (7.0)	1,102 (7.4)
45–50	446 (6.1)	432 (5.2)	878 (5.7)
50+	1,071 (14.7)	700 (9.4)	1,771 (12.1)
Zone			
North	839 (13.4)	868 (12.2)	1,707 (12.8)
Central East	1,262 (19.3)	1,315 (16.7)	2,577 (18.1)
Central West	981 (22.1)	1,082 (22.0)	2,063 (22.0)
Lilongwe City	294 (4.8)	421 (5.7)	715 (5.2)
South East	1,373 (17.7)	1,875 (20.0)	3,248 (18.9)
South West	1,356 (17.9)	1,631 (18.7)	2,987 (18.3)
Blantyre City	327 (4.7)	371 (4.7)	698 (4.7)
Highest level of school respondent ever attended			
No education	429 (6.4)	845 (11.2)	1,274 (8.7)
Primary	3,793 (58.2)	4,956 (65.2)	8,749 (61.6)
Secondary	1,921 (30.6)	1,574 (21.0)	3,495 (25.9)
More than secondary	284 (4.8)	184 (2.5)	468 (3.7)
Residence			
Urban	993 (16.6)	1,267 (15.8)	2,260 (16.2)
Rural	5,439 (83.4)	6,296 (84.2)	11,735 (83.8)
Marital status			
Never married	1,615 (26.0)	824 (12.7)	2,439 (19.5)
Married or living together	4,526 (69.7)	5,884 (76.5)	10,410 (73.0)
Divorced or separated	273 (4.1)	777 (9.9)	1,050 (6.9)
Widowed	15 (0.2)	73 (1.0)	88 (0.6)
Wealth quintile			
Lowest	1,096 (17.7)	1,399 (19.3)	2,495 (18.5)
Second	1,346 (20.7)	1,566 (20.8)	2,912 (20.7)
Middle	1,290 (19.6)	1,555 (19.9)	2,845 (19.8)
Fourth	1,425 (21.8)	1,528 (19.7)	2,953(20.8)
Highest	1,274 (20.2)	1,514 (20.2)	2,788 (20.2)

### Awareness of PrEP and factors associated with PrEP awareness

Overall, 15.0% (95% CI: 14.2–15.9%) of the participants reported being aware of PrEP (Table 2). Awareness was higher in males (aOR: 1.3, 95% CI: 1.2–1.5), participants with secondary (aOR: 1.5, 95% CI: 1.2-2.0) and post-secondary education (aOR: 3.4, 95% CI: 2.4–4.9) and those from the fourth (aOR: 1.2, 95% CI: 1.0-1.5) and highest wealth quintiles (aOR: 1.6, 95% CI: 1.2-2.0) compared to females, those with no education and those from the lowest wealth quintile, respectively. There were also differences in awareness by geographic residence (zone) of the participants.

**Table 2:** Factors associated with oral PrEP awareness among sexually active HIV-negative participants from MPHIA 2020–2021 survey

Characteristic	Aware of oral PrEP (N = 13,995)				
	Weighted % (95% CI)	Unadjusted Odds Ratio (95% CI)	p-value	Adjusted Odds Ratio (95% CI)	p-value
Total	15.0 (14.2–15.9)	-	-	-	-
Gender					
Female	12.5 (11.6–13.4)	Ref	-	Ref	-
Male	17.3 (16.2–18.5)	1.5 (1.3–1.6)	< 0.001	1.3 (1.2–1.5)	< 0.001*
Zone					
North	18.2 (15.5–21.3)	Ref	-	Ref	-
Central East	13.1 (11.4–15.1)	0.7 (0.5–0.9)	0.005	0.8 (0.6-1.0)	0.023*
Central West	11.1 (9.2–13.3)	0.6 (0.4–0.7)	< 0.001	0.7 (0.5–0.9)	0.002*
Lilongwe City	19.7 (15.9–24.1)	1.1 (0.8–1.5)	0.556	0.6 (0.4–0.9)	0.008*
South East	14.8 (13.2–16.5)	0.8 (0.6-1.0)	0.042	0.9 (0.7–1.1)	0.442
South West	16.1 (14.5–17.9)	0.9 (0.7–1.1)	0.196	1.0 (0.8–1.2)	0.910
Blantyre City	22.5 (18.2–27.6)	1.3 (0.9–1.8)	0.118	0.7 (0.5-1.0)	0.051
Age in years					
15–19	13.5 (11.6–15.5)	Ref	-	Ref	-
20–24	15.2 (13.6–16.8)	1.2 (0.9–1.4)	0.195	1.1 (0.9–1.4)	0.306
25–29	16.5 (14.9–18.3)	1.3 (1.0-1.6)	0.027*	1.2 (0.9–1.6)	0.135
30–34	16.8 (14.8–19.1)	1.3 (1.0-1.6)	0.026	1.3 (1.0-1.7)	0.07
35–39	15.3 (13.3–17.6)	1.2 (0.9–1.4)	0.185	1.2 (0.9–1.6)	0.174
40–44	16.5 (14.2–19.1)	1.3 (1.0-1.6)	0.063	1.3 (0.8–1.8)	0.055
45–50	12.9 (10.8–15.4)	0.9 (0.7–1.2)	0.681	1.1 (0.8–1.4)	0.699
50+	11.6 (10.1–13.3)	0.8 (0.7–1.1)	0.131	1.0 (0.7–1.3)	0.851
Highest level of school respondent ever attended					
No education	10.6 (8.7–12.8)	Ref	-	Ref	-
Primary	11.8 (11.0-12.6)	1.1 (0.9–1.4)	0.257	1.0 (0.8–1.2)	0.953
Secondary	20.4 (18.8–22.1)	2.2 (1.7–2.8)	< 0.001	1.5 (1.2-2.0)	0.002*
More than secondary	40.6 (35.5–45.9)	5.8 (4.2–7.9)	< 0.001	3.4 (2.4–4.9)	< 0.001*
Urban/rural area Indicator					
Rural	13.5 (12.7–14.3)	Ref	-	Ref	-
Urban	22.6 (20.1–25.3)	1.9 (1.6–2.2)	< 0.001	1.3 (1.0-1.7)	0.064
Marital status					
Never married	18.3 (16.6–20.3)	Ref	-	Ref	-
Married or living together	14.2 (13.3–15.1)	0.7 (0.6–0.8)	< 0.001	0.9 (0.7–1.1)	0.261
Divorced or separated	13.8 (11.5–16.3)	0.7 (0.6–0.9)	0.006	0.9 (0.7–1.2)	0.512
Widowed	12.0 (6.4–21.3)	0.6 (0.3–1.2)	0.158	0.8 (0.4–1.6)	0.524
Wealth quintile					
Lowest	10.6 (9.2–12.1)	Ref	-	Ref	-
Second	11.5 (10.2–12.9)	1.1 (0.9–1.3)	0.316	1.0 (0.9–1.2)	0.717
Middle	13.5 (12.1–14.9)	1.3 (1.1–1.6)	0.007	1.2 (1.0-1.4)	0.124
Fourth	15.3 (13.9–16.9)	1.5 (1.3–1.8)	< 0.001	1.2 (1.0-1.5)	0.035
Highest	23.7 (21.6–25.9)	2.6 (2.1–3.2)	< 0.001	1.6 (1.2-2.0)	0.001

* Statistically significant at p-value = 0.05

### Willingness to use and factors associated with willingness to use PrEP

The proportion of people who were willing to use PrEP was 73.0% (95% CI: 71.8–74.1%) (Table 3). In the adjusted analysis (Table 3), being male (aOR: 1.2; 95% CI: 1.1–1.3) and having more than one sexual partner in the prior 12 months (aOR: 1.7; 95% CI: 1.4–1.9) were associated with higher willingness to use PrEP than being female and having only one sexual partner, respectively. Older participants were less willingness to take PrEP than younger particpants. Increasing age was associated with declining interest in PrEP. Willingness to use PrEP was higher among persons who reported being tested for HIV in the previous 12 months than among those who had never tested.

**Table 3:** Factors associated with willingness to use oral PrEP among sexually active adult MPHIA 2020 survey participants

Characteristic	Willing to use oral PrEP				
	Weighted % (95% CI)	Unadjusted Odds Ratio (95% CI)	P-value	Adjusted Odds Ratio (95% CI)	P-value
Total	73.0 (71.8–74.1)	-	-	-	-
Gender					
Female	71.2 (69.8–72.6)	Ref	-	Ref	-
Male	74.7 (73.3–76.0)	1.2 (1.1–1.3)	< 0.001	1.2 (1.1–1.3)	0.001*
Zone					
North	78.7 (75.3–81.7)	Ref	-	Ref	-
Central East	73.9 (71.5–76.1)	0.8 (0.6-1.0)	0.022	0.7 (0.6–0.9)	0.013*
Central West	67.6 (64.9–70.2)	0.6 (0.5–0.7)	< 0.001	0.6 (0.4–0.7)	< 0.001*
Lilongwe City	68.8 (65.6–71.8)	0.6 (0.5–0.8)	< 0.001	0.6 (0.5–0.9)	0.008*
South East	77.0 (74.8–79.2)	0.9 (0.7–1.1)	0.394	0.9 (0.7–1.1)	0.273
South West	71.4 (68.6–74.1)	0.7 (0.5–0.9)	0.002	0.7 (0.5–0.8)	0.001*
Blantyre City	73.5 (69.4–77.2)	0.8 (0.6-1.0)	0.038	0.8 (0.6–1.2)	0.300
Age in years					
15–19	77.6 (75.2–79.9)	Ref	-	Ref	-
20–24	74.8 (72.7–76.7)	0.9 (0.7-1.0)	0.043*	0.8 (0.7-1.0)	0.032
25–29	75.3 (73.2–77.4)	0.9 (0.7–1.1)	0.149	0.9 (0.7–1.1)	0.192
30–34	71.4 (69.1–73.6)	0.7 (0.6–0.8)	< 0.001	0.7 (0.6–0.9)	0.004*
35–39	72.1 (69.7–74.3)	0.7 (0.6–0.9)	0.001*	0.8 (0.6–0.9)	0.016*
40–44	72.6 (69.3–75.7)	0.8 (0.6–0.9)	0.012	0.8 (0.6–1.1)	0.113
45–49	70.3 (67.1–73.4)	0.7 (0.6–0.8)	< 0.001	0.7 (0.6–0.9)	0.018*
50+	65.3 (62.6–67.9)	0.5 (0.5–0.6)	< 0.001*	0.6 (0.5–0.7)	< 0.001*
Highest level of school attended					
No education	67.0 (63.6–70.1)	Ref	-	Ref	-
Primary	73.2 (71.9–74.5)	1.3 (1.2–1.6)	< 0.001	1.1 (1.0-1.3)	0.117
Secondary	75.0 (73.3–76.7)	1.5 (1.3–1.7)	< 0.001	1.2 (1.0-1.4)	0.074
More than secondary	68.1 (63.3–72.6)	1.1 (0.8–1.4)	0.685	1.0 (0.7–1.3)	0.824
Residence					
Urban	72.0 (69.7–74.1)	Ref	-	Ref	-
Rural	73.2 (71.8–74.4)	1.1 (0.9–1.2)	0.346	0.9 (0.7–1.2)	0.439
Marital status					
Never married	77.0 (74.7–79.1)	Ref	-	Ref	-
Married or living together	71.4 (70.2–72.6)	0.7 (0.7–0.9)	< 0.001	1.0 (0.8–1.2)	0.834
Divorced or separated	77.8 (74.8–80.5)	1.0 (0.9–1.3)	0.635	1.2 (1.0-1.6)	0.084
Widowed	74.7 (63.1–83.6)	0.9 (0.5–1.5)	0.628	1.5 (0.8–2.8)	0.153
Wealth quintile					
Lowest	71.8 (69.2–74.2)	Ref-	-	Ref	-
Second	73.8 (71.8–75.7)	1.1 (1.0-1.3)	0.113	1.0 (0.9–1.2)	0.661
Middle	74.0 (71.8–76.1)	1.1 (1.0-1.3)	0.150	1.1 (0.9–1.3)	0.487
Fourth	73.2 (71.1–75.1)	1.1 (0.9–1.3)	0.366	1.0 (0.9–1.2)	0.896
Highest	72.0 (69.6–74.2)	1.0 (0.9–1.2)	0.900	0.9 (0.8–1.1)	0.483
Number of sexual partners					
1	71.1 (69.9–72.3)	Ref	-	Ref	-
2 or more	82.2 (80.2–84.0)	1.9 (1.6–2.1)	< 0.001	1.7 (1.4–1.9)	< 0.001*
Previous HIV test ≤ 12 months					
Never tested	70.9 (68.0-73.7)	Ref	-	Ref	-
Yes	74.7 (73.2–76.1)	1.4 (1.2–1.7)	0.001	1.3 (1.1–1.5)	0.008*
No	72.0 (70.2–73.5)	1.2 (1.0-1.4)	0.096	1.1 (0.9–1.3)	0.179

* Statistically significant at p-value = 0.05

## Discussion

In this nationally representative survey of adult participants within a low-income setting, we found that PrEP awareness was low but willingness to use PrEP to prevent HIV was high among HIV-negative sexually active respondents. There were disparities in awareness of PrEP by gender, geographic residence, level of education, urbanicity, and marital and wealth status. Additionally, we identified several factors associated with the willingness to use PrEP which may be important in informing strategies for PrEP delivery to further reduce HIV transmission.

From the analysis, although general awareness for PrEP was low (15%), there were notable disparities in PrEP awareness by level of education, urban residence, and wealth status. The low awareness of PrEP among those less educated and from poorer households and rural areas in our analysis may signify that the current HIV preventive strategies may not be adequately reaching these populations. Up to 84% of Malawians reside in the rural areas [15] and tend to be less educated and less wealthy. Previous studies have reported print media and internet websites as important sources of PrEP information [7, 16]. These sources may be inaccessible to poor and illiterate individuals. From MPHIA 2020 result [14], the prevalence of HIV was highest among those with no education and decreased with increasing level of education. To address these disparities, there may be need to design and utilize segmented social marketing strategies to disseminate HIV prevention information, accounting for measures such as level of education and access to messaging platforms to reach underserved populations [17]. The design of the strategies may be informed by both qualitative (understanding beliefs, attitudes, influences, and knowledge) and quantitative methods. The implementation of prevention strategies may require continued monitoring and evaluation to identify and address any emerging or remaining factors affecting reach and utilization.

The willingness to use PrEP was higher among participants who reported high risk behavior but lower in older than young people. Those who reported having sex with more than one partner were more willing to use PrEP. Both perceived risk and actual risk have been previously shown to increase willingness to use PrEP in previous studies [10, 18–20]. This specific willingness to take PrEP by those with a perceived high risk may support the need to improve access to PrEP to all persons. A cluster randomized trial in Kenya reported no difference in PrEP uptake and HIV incidence between risk-based and universal PrEP delivery strategies in pregnant women [21]. The universal approach was reported to be less time-consuming and did not expose clients to uncomfortable discussions, hence being simpler [22]. Risk is only known by individuals themselves; using strategies that target specific populations may therefore be unfavorable to those with unremarkable ongoing risk or require further probing to assess the “risk”. For young people, consideration for PrEP delivery in schools, for those attending schools, and within other social activities, for those outside school may increase their access to PrEP.

Those who had accessed HTS in the prior 12 months were more aware of PrEP (Table 2) and may therefore have been more willing to use PrEP. Also, those who reported accessing HIV testing services (HTS) in the prior 12 months were more willing to use PrEP. The high willingness for those already utilizing and accessing HTS provides an opportunity to integrate PrEP services and increase access to PrEP which was low in the survey. A previous study in AGYW from Kenya and South Africa demonstrated an increase in demand when PrEP was integrated in primary care and reproductive health services [23].

Both awareness of and willingness to use PrEP were lower in female than male participants in the survey. PrEP awareness was however lower in younger people [15–19] while interest was higher in this group. PrEP awareness has been identified as an important factor for the willingness to use PrEP by women in other studies [24–26]. Several factors may be associated with a low PrEP awareness in females including the low level of education in female participants demonstrated in this study (Table 1) and discussed above. Empowering females by promoting girls’ education and school participation may improve awareness and utilization of HIV interventions. Incorporating school-based approaches to integrate HIV prevention messaging, including PrEP information and delivery for both boys and girls, may promote positive gender norms, health awareness and uptake of preventive measures [27].

It is worth acknowledging the strengths and limitations of these results. An important strength is the large sample size which included a nationally representative sample of the adult population. To our knowledge, this is the first analysis of PrEP awareness and interest in the general population in Malawi (outside of “priority populations”). One limitation may stem from social desirability bias where respondents provide a response which they perceive to be pleasing or acceptable to the interviewer or others. In this context, some participants would have indicated their willingness to take PrEP because they perceived that response as more acceptable. Another limitation is the measure of “willingness” to use PrEP which, for most participants, was measured only after they just learned about PrEP and, as such, may not reflect the uptake, adherence, and persistence of PrEP. Finally, the measure of “willingness” as an indication of intention, may not accurately reflect behavior (i.e., actual use of PrEP) which may need to account for other considerations. However, there were appreciable trends observed from the responses which may inform further research and strategies for PrEP delivery.

## Conclusion

Strategies to reach sexually active population with PrEP information may increase awareness, willingness to use, and uptake of PrEP for HIV prevention. In low-income settings, prioritizing access to education and attainment may improve health literacy and access to information and interventions to contribute to disease prevention and control. Improving access of PrEP, by making it available, acceptable, and affordable, may increase uptake of PrEP among all people at risk of HIV acquisition. Integrating PrEP within routine health services (e.g., reproductive health, family planning, antenatal and postnatal care), may improve availability of PrEP among sexually active individuals.

## Data Availability

The datasets supporting the conclusions of this article are available at ICAP website: https://phia-data.icap.columbia.edu/.

## List of Abbreviations

AGYW: Adolescent girls and young women
aOR: Adjusted odds ratio
CDC: US Centers for Disease Control and Prevention
CI: Confidence interval
HTS: HIV testing services
MPHIA: Malawi Population-based HIV Impact Assessment
MSM: Men who have sex with men
OR: Odds ratio
PEPFAR: President’s Emergency Plan for AIDS Relief
PrEP: Pre-exposure prophylaxis
VMMC: Voluntary medical male circumcision
WHO: World Health Organization
